# Does fluoxetine reduce apathetic and depressive symptoms after stroke? An analysis of the Efficacy oF Fluoxetine—a randomized Controlled Trial in Stroke trial data set

**DOI:** 10.1177/17474930221124760

**Published:** 2022-09-19

**Authors:** Jonathan Tay, EFFECTS Trial Collaboration, Björn Mårtensson, Hugh S Markus, Erik Lundström

**Affiliations:** 1 Stroke Research Group, Department of Clinical Neurosciences, University of Cambridge, Cambridge, UK; 2 Department of Clinical Neuroscience, Karolinska Institute, Stockholm, Sweden; 3 Department of Neuroscience, Neurology, Uppsala University, Uppsala, Sweden

**Keywords:** Apathy, depression, SSRI, clinical, Stroke, cognition

## Abstract

**Objective:**

Apathy is a common and disabling symptom after stroke with no proven treatments. Selective serotonin reuptake inhibitors are widely used to treat depressive symptoms post-stroke but whether they reduce apathetic symptoms is unknown. We determined the effect of fluoxetine on post-stroke apathy in a post hoc analysis of the EFFECTS (Efficacy oF Fluoxetine—a randomized Controlled Trial in Stroke) trial.

**Methods:**

EFFECTS enrolled patients ⩾18 years between 2 and 15 days after stroke onset. Participants were randomly assigned to receive oral fluoxetine 20 mg once daily or matching placebo for 6 months. The Montgomery–Åsberg Depression Rating Scale (MADRS) was administered at baseline and 6 months. Individual items on this scale were divided into those reflecting symptoms of apathy and depression. Symptoms were compared between fluoxetine and placebo groups.

**Results:**

Of 1500 participants enrolled, complete MADRS data were available for 1369. The modified intention-to-treat population included 681 patients in the fluoxetine group and 688 in the placebo group. Confirmatory factor analysis revealed that apathetic, depressive, and anhedonic symptoms were dissociable. Apathy scores increased in both fluoxetine and placebo groups (both p ⩽ 0.00001). In contrast, fluoxetine was associated with a reduction in depressive scores (p = 0.002)

**Conclusion:**

Post-stroke apathetic and depressive symptoms respond differently to fluoxetine treatment. Our analysis suggests fluoxetine is ineffective in preventing post-stroke apathy.

## Introduction

Apathy occurs in one out of three patients after stroke.^1^ It describes a reduction in goal-directed activity in the cognitive, behavioral, emotional, or social domains of a patient’s life. Despite its frequency, apathy is clinically under-recognized, and there are no proven drug treatment approaches.

Antidepressants, such as selective serotonin reuptake inhibitors (SSRIs), are often used to treat post-stroke depression. Whether SSRIs are also effective in apathy remains uncertain.^1^ Despite shared symptoms, post-stroke apathy and depression are dissociable syndromes. Negative emotionality is a key characteristic of depression that distinguishes it from apathy. Depressed patients may present with pessimism and hopelessness, while those with apathy show lack of emotional distress. Recent studies suggest apathy and depression have different neuroanatomical correlates with white matter track damage and subsequent complex network disruption underlying apathy, but not depression.^1,2^ This suggests they might respond differently to therapeutic interventions.

We determined whether fluoxetine reduced apathy in a post hoc analysis of data from the Efficacy oF Fluoxetine, a randomized Controlled Trial in Stroke (EFFECTS) trial.

## Methods

### Participants

EFFECTS was a randomized, double-blind, placebo-controlled clinical trial conducted in 35 hospital centers in Sweden.^3^ Eligible participants were adults (age ⩾18 years) with a diagnosis of acute stroke within the previous 2–15 days, brain imaging consistent with ischemic or hemorrhagic stroke, and persisting neurological deficit. Exclusion criteria included depression or antidepressant use; fluoxetine contraindication; unlikely to be available for follow-up; unlikely to survive until follow-up; enrollment in another clinical trial of an investigational medical product or device; women if pregnant, breast-feeding, or of child-bearing age and not using contraception. Apathy was not an exclusion criteria.

Participants were randomized via a secure, centralized web-based system using a minimization algorithm that assigned participants to fluoxetine 20 mg once daily (od) or placebo one od for 6 months in a 1:1 ratio.^3^ Placebo capsules were visually identical to fluoxetine capsules. Patients were followed-up at 6 months by postal questionnaire or telephone from the trial coordinating center. If patients were unable to complete questionnaires, assistance was sought from their next of kin or carer. The participant, care provider, investigator, and outcome assessor remained masked to allocated trial treatment until completion of the study. The design, methods, and primary results have been published.^3^

EFFECTS was approved by a medical ethics committee in Stockholm (reference 2013/1265-31/2) and the Swedish Medical Agency (reference 5.1-2014-43006). The study was registered in the EU Clinical Trials Register (clinicaltrialsregister.eu; EudraCT no. 2011-006130-16) and at ClinicalTrials.gov (https://clinicaltrials.gov/ct2/show/NCT02683213). All participants provided written informed consent.

### Clinical measures and confirmatory factor analysis

Stroke severity was quantified using the National Institutes of Health Stroke Scale (NIHSS).^4^ Apathy and depression were assessed using the Montgomery–Åsberg Depression Rating Scale (MADRS).^5^ The MADRS has 10 items, each graded from 0 (no symptoms) to 6 (most severe symptoms). Item 7 on the MADRS (lassitude) assesses difficulty in starting and completing everyday tasks, and was determined to be a measure of apathy. Item 8 (inability to feel) assesses anhedonia, and given theoretical overlap with apathy,^6^ was investigated separately to other depressive symptoms. The remaining eight items were used as a measure of depression. We conducted confirmatory factor analysis (CFA) to validate our use of these measures.

CFA quantitatively tests whether a hypothesized scale structure matches the observed data.^7^ In the context of this study, we tested the hypothesis that separating the MADRS into apathy and depression subscales would yield a better fit to the observed data compared to taking all the items on the MADRS to represent a unitary depressive construct. Previous studies have found support for a multi-factorial structure of the MADRS that included apathy in stroke patients,^8^ but not in Parkinson’s disease.^9^ Importantly, CFA alone does not assess whether patients have apathy or depression per se, but rather identify whether patterns of responding on certain items are correlated.

We tested this by comparing nested models that used the same variables, but different hypothesized factor structures.^10^ The initial baseline model was a one-factor model of depression, where all items on the MADRS loaded onto a single depression construct. The second model was a three-factor model that separately assessed apathy, depression, and anhedonia. Anhedonia was separated from other symptoms of depression due to its theoretical overlap with apathy. Apathy was assessed using item 7, anhedonia using item 8, and depression using the remaining items on the MADRS. Models were compared using the Akaike information criterion (AIC) and Bayesian information criterion (BIC). For both measures, lower values indicate better fitting models, with a difference of 10 being indicative of a significantly different model.^11^

All models were fitted using default arguments to the cfa() function in laavan 0.6-7.^12^ Although the Likert-type responses on the MADRS suggest that items should be treated as ordinal variables, simulation studies have shown that maximum likelihood estimation yields similar results to categorical estimation methods when applied to variables with six to seven categories.^13^ As items on the MADRS were graded on a 7-point scale, we opted to treat items as continuous variables and use maximum likelihood to estimate CFA models.

### Statistical analyses

Statistics were calculated using R 4.0.4. All tests were two-tailed with ɑ = 0.05. As this was a post hoc analysis, no power calculations were conducted; power calculations for the main trial have been published.^7^

Mann–Whitney *U* and Wilcoxon signed-rank tests were used to compare apathy and depression between-groups (fluoxetine vs placebo) and within-groups (baseline to 6 months). Analyses were repeated in the following pre-specified subgroups: age (⩽70 or >70), sex, stroke type (ischemic or hemorrhagic), ischemic stroke subtype classified using modified Trial of Org 10172 in Acute Stroke Treatment (TOAST) criteria,^14^ NIHSS (⩽5 or >5), and medication adherence (taken 7 days a week or not). p values across all subgroup analyses were corrected for multiple comparisons using the false discovery rate (FDR).

To further validate longitudinal comparisons, patients were grouped into whether they had apathy symptoms (i.e. item 7 score >0) at baseline and at 6 months. McNemar’s test was applied to the resulting 2 × 2 contingency table to determine if the proportion of patients endorsing apathy symptoms changed after fluoxetine treatment. This test was conducted in both fluoxetine and placebo groups. This analysis was also repeated for anhedonia symptoms (item 8 score >0).

### Data availability statement

Anonymized data supporting the findings of the trial are available to researchers upon reasonable request to the corresponding author (EL; erik.lundstrom@neuro.uu.se) following receipt of a written application and proposal for use of the data, approval by the EFFECTS trial Steering Committee, and establishment of a data sharing agreement.

## Results

### Population characteristics

**Figure 1.**
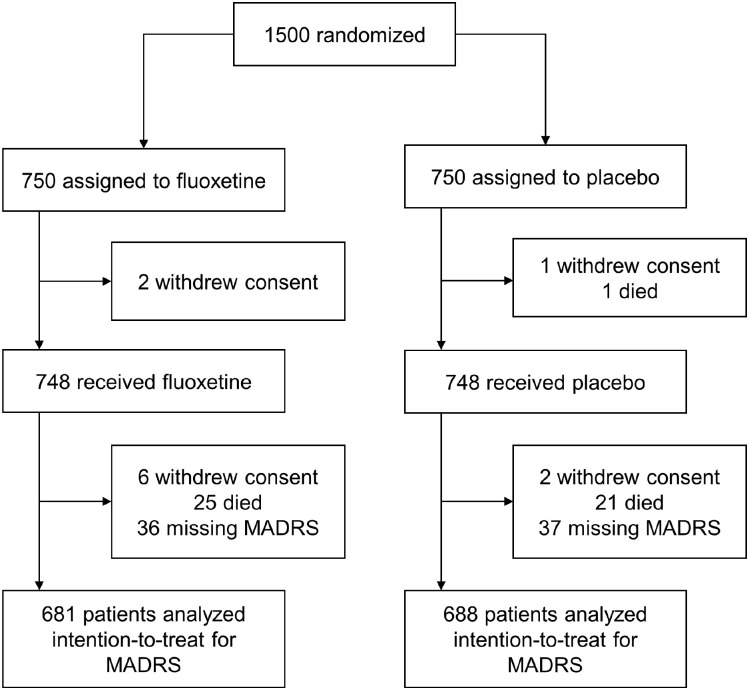
Consort flow diagram.

Recruitment started 20 October 2014 and ended 28 June 2019. One thousand five-hundred participants were randomized to fluoxetine (n = 750) or placebo (n = 750). Of these, 1369 had complete MADRS scores at 6 months (fluoxetine = 681 and placebo = 688) and were included in the modified intention-to-treat (ITT) analysis (Figure 1). Excluded patients were older and had higher NIHSS scores (Supplementary Table 1). Baseline characteristics were well balanced between both groups, although the fluoxetine group showed higher apathy scores at baseline (Table 1).

**Table 1.** Baseline characteristics of the modified intention-to-treat population in EFFECTS.

	Fluoxetine (n = 681)	Placebo (n = 688)	p
Age, mean (SD)	70 (11.2)	71.4 (10.4)	0.37
Sex, female, n (%)	270 (39.6)	253 (36.8)	0.32
NIHSS, median (IQR)	3.0 (2–6)	3.0 (2–6)	0.61
Stroke type			0.55
Ischemic, n (%)	599 (88.0)	595 (86.5)	
Hemorrhagic, n (%)	82 (12.0)	91 (13.2)	
Ischemic stroke cause*			0.27
Large artery disease, n (%)	95 (14.0)	81 (11.8)	
Small-vessel disease, n (%)	199 (29.2)	196 (28.5)	
Cardioembolism, n (%)	120 (17.6)	134 (19.5)	
Other, n (%)	25 (3.7)	14 (2.0)	
Unknown or uncertain, n (%)	164 (24.1)	172 (25.0)	
MADRS variables			
Total score, mean (SD)	2.8 (3.6)	2.7 (3.0)	0.87
Apathy, mean (SD)	0.4 (0.8)	0.3 (0.7)	0.007
Depression, mean (SD)	2.1 (2.7)	2.1 (2.3)	0.71
Anhedonia, mean (SD)	0.1 (0.4)	0.1 (0.3)	0.28

NIHSS: National Institute of Health Stroke Scale; MADRS: Montgomery–Åsberg Depression Rating Scale.

* Assessed using modified TOAST criteria.

### CFA

**Figure 2.**
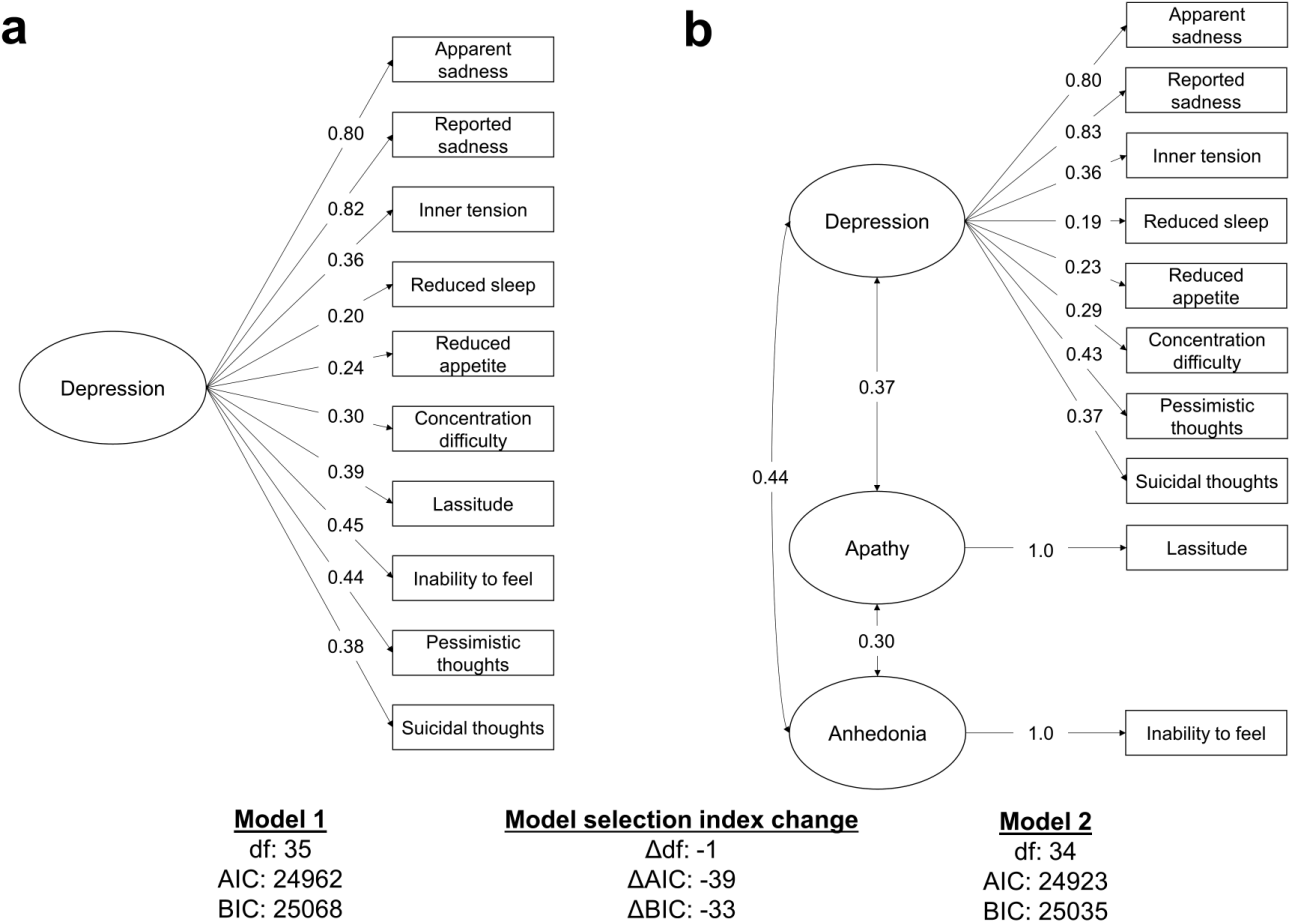
Confirmatory factor analysis of the Montgomery–Åsberg Depression Rating Scale. (a) One-factor model of depression and (b) three-factor model assessing apathy, depression, and anhedonia. For both indices, lower values indicate a better fit to the data, with differences over 10 being considered significant. The three-factor model demonstrated lower AIC and BIC values, suggesting it was a better fit to the data. Single-headed arrows indicate the factor loadings while double-headed arrows indicate the correlations. All paths are significant at p < 0.05. AIC: Akaike information criterion; BIC: Bayesian information criterion.

Comparison of CFA models revealed that a three-factor model that separately assessed apathetic, depressive, and anhedonic symptoms was a better fit to baseline MADRS data than a single-factor depression model (Figure 2). The three-factor model demonstrated lower AIC and BIC values compared to the one-factor model, with differences greater than 30, indicating that assessing the symptoms separately better explained the observed data. All factors showed moderately strong inter-factor correlations (β = 0.30–0.44).

### Comparisons between fluoxetine and placebo

Depression scores decreased in the fluoxetine group (baseline = 2.11 (2.67); 6 months = 1.75 (2.41), p = 0.002) but not in the placebo group (baseline = 2.07 (2.34); 6 months = 2.09 (3.14), p = 0.19). In contrast, apathetic scores increased in both fluoxetine (baseline = 0.38 (0.84); 6 months = 0.63 (1.08)) and placebo (baseline = 0.29 (0.75); 6 months = 0.52 (1.03)) groups (both p ⩽ 0.00001). There appeared to be a trend toward increasing anhedonia in both groups (fluoxetine baseline = 0.10 (0.39), 6 months = 0.12 (0.41); placebo baseline = 0.07 (0.34), 6 months = 0.13 (0.50)), but this was only significant in the placebo group (p = 0.01). There was no change in total MADRS score between baseline and 6 months in either group.

We analyzed subgroups to determine factors related to change in depressive and apathetic scores between baseline and 6 months. Results were largely consistent with main longitudinal results (Table 2 and Supplementary Table 2). Apathetic symptoms increased in most subgroups except the following: female (fluoxetine), hemorrhagic stroke (placebo), ischemic stroke patients with large artery disease, cryptogenic, or other strokes (fluoxetine and placebo), NIHSS >5 (placebo), and non-adherent (placebo). Decreased depressive symptoms in patients on fluoxetine were observed in the following subgroups: age ⩽70 years, ischemic stroke, ischemic stroke patients with small-vessel disease (SVD), and NIHSS ⩽5.

**Table 2.** Longitudinal subgroup comparisons within fluoxetine and placebo groups in EFFECTS.

		Fluoxetine				Placebo			
		n	Baseline	6 months	P_FDR_	n	Baseline	6 months	P_FDR_
Age									
>70	Total	405	2.66	2.82	0.85	383	2.72	2.95	0.61
	Apathy	405	0.38	0.62	0.0008	383	0.3	0.49	0.03
	Depression	405	1.99	1.84	0.45	383	2.12	2.12	0.36
	Anhedonia	405	0.09	0.13	0.28	383	0.08	0.11	0.58
⩽70	Total	276	3.07	2.51	0.18	303	2.61	3.08	0.61
	Apathy	276	0.38	0.63	0.007	303	0.29	0.56	0.003
	Depression	276	2.29	1.61	0.008	303	2.01	2.07	0.86
	Anhedonia	276	0.12	0.11	0.86	303	0.07	0.16	0.04
Sex									
Female	Total	270	3.07	2.79	0.45	253	2.48	2.83	0.66
	Apathy	270	0.4	0.54	0.15	253	0.25	0.51	0.009
	Depression	270	2.3	1.87	0.08	253	1.88	1.94	0.92
	Anhedonia	270	0.11	0.13	0.85	253	0.1	0.15	0.56
Male	Total	411	2.66	2.63	0.93	433	2.79	3.11	0.83
	Apathy	411	0.37	0.68	0.00002	433	0.32	0.53	0.009
	Depression	411	1.99	1.67	0.1	433	2.18	2.19	0.37
	Anhedonia	411	0.09	0.12	0.58	433	0.06	0.12	0.08
Stroke type									
Ischemic	Total	599	2.82	2.64	0.61	595	2.62	2.92	0.92
	Apathy	599	0.39	0.62	0.0002	595	0.28	0.5	0.0003
	Depression	599	2.1	1.7	0.02	595	2.05	2.05	0.41
	Anhedonia	599	0.1	0.13	0.56	595	0.07	0.13	0.06
Hemorrhagic	Total	82	2.88	3.12	0.85	91	3.04	3.54	0.83
	Apathy	82	0.34	0.7	0.03	91	0.41	0.63	0.28
	Depression	82	2.22	2.07	0.56	91	2.24	2.38	0.93
	Anhedonia	82	0.09	0.1	1	91	0.11	0.16	0.73
Ischemic stroke type									
Large artery disease	Total	95	2.56	3.08	0.18	81	2.91	3.73	0.56
	Apathy	95	0.36	0.68	0.09	81	0.37	0.56	0.53
	Depression	95	1.95	1.93	0.94	81	2.27	2.62	0.85
	Anhedonia	95	0.07	0.22	0.19	81	0.07	0.25	0.16
Small-vessel disease	Total	199	2.88	2.61	0.41	196	2.07	2.64	0.25
	Apathy	199	0.39	0.65	0.02	196	0.22	0.45	0.04
	Depression	199	2.21	1.69	0.04	196	1.66	1.9	0.67
	Anhedonia	199	0.1	0.1	1	196	0.04	0.11	0.09
Cardioembolism	Total	120	2.71	2.19	0.28	134	2.87	3.37	0.79
	Apathy	120	0.29	0.58	0.01	134	0.32	0.6	0.03
	Depression	120	2.02	1.39	0.06	134	2.17	2.34	0.83
	Anhedonia	120	0.12	0.07	0.61	134	0.12	0.15	0.85
Other	Total	25	2.8	3.04	0.99	14	4.14	2.93	0.45
	Apathy	25	0.16	0.48	0.32	14	0.14	0.07	1
	Depression	25	2.44	2.32	0.85	14	3.36	2.43	0.61
	Anhedonia	25	0.12	0.08	0.93	14	0.14	0	0.58
Unknown	Total	164	2.52	2.61	0.88	172	2.77	2.42	0.04
	Apathy	164	0.4	0.52	0.33	172	0.28	0.44	0.2
	Depression	164	1.79	1.76	0.99	172	2.15	1.67	0.009
	Anhedonia	164	0.08	0.12	0.53	172	0.08	0.08	0.97
NIHSS									
5	Total	211	2.38	2.7	0.56	188	2.74	2.77	0.85
	Apathy	211	0.3	0.7	0.0002	188	0.32	0.45	0.34
	Depression	211	1.84	1.66	0.53	188	2.05	1.95	0.61
	Anhedonia	211	0.05	0.12	0.2	188	0.1	0.12	0.85
⩽5	Total	470	3.02	2.69	0.2	498	2.65	3.09	0.93
	Apathy	470	0.42	0.59	0.009	498	0.28	0.55	0.0003
	Depression	470	2.23	1.79	0.02	498	2.08	2.15	0.56
	Anhedonia	470	0.12	0.12	0.98	498	0.07	0.14	0.03
Medication adherence									
Adherent	Total	542	2.78	2.56	0.41	550	2.74	3.2	0.86
	Apathy	542	0.39	0.62	0.0003	550	0.3	0.56	0.0002
	Depression	542	2.07	1.63	0.007	550	2.13	2.21	0.56
	Anhedonia	542	0.1	0.12	0.66	550	0.08	0.15	0.05
Not adherent	Total	138	3.01	3.22	0.85	135	2.42	2.24	0.67
	Apathy	138	0.36	0.62	0.01	135	0.28	0.36	0.58
	Depression	138	2.28	2.23	0.94	135	1.87	1.65	0.62
	Anhedonia	138	0.09	0.12	0.79	135	0.05	0.07	0.86

FDR: false discovery rate; NIHSS: National Institutes of Health Stroke Scale.

**Figure 3.**
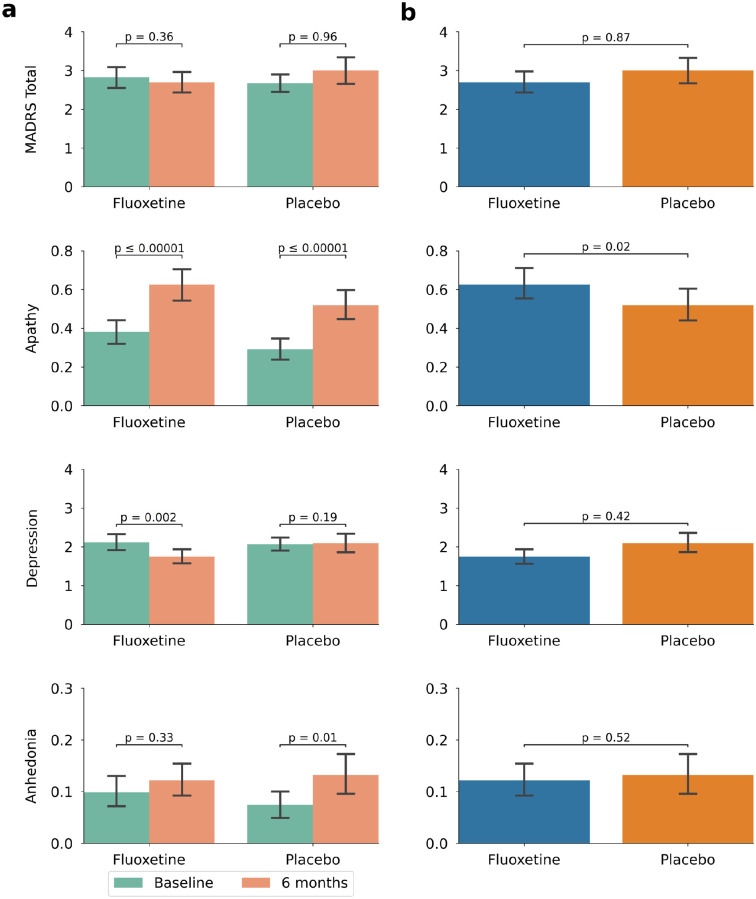
Results in the modified intention-to-treat population. All measures were derived from the Montgomery–Åsberg Depression Rating Scale. Note that scale axes have been truncated for clarity; the maximum scores are 6 for apathy and 48 for depression and 6 for anhedonia. (a) Within-group comparisons between baseline and 6 months. Total score did not change in either group. Apathetic symptoms increase in both groups, depressive symptoms decrease in the fluoxetine group, and anhedonic symptoms increase in the placebo group. (b) Comparisons between the fluoxetine and placebo groups at 6 months, with the fluoxetine group showing higher apathetic symptoms.

Between-group comparisons between fluoxetine and placebo at 6 months revealed no differences in MADRS total score, depressive, or anhedonic symptoms, although apathetic symptoms were elevated in the fluoxetine group (Figure 3(b)). Due to these negative treatment effects, no between-group subgroup analysis was performed.

### Comparison of proportions

In the fluoxetine group, 7.5% of patients had apathy (defined as item 7 score <1) at baseline and follow-up, 14.4% had apathy at baseline, but not at follow-up, 16.3% had no apathy at baseline, but had apathy at follow-up, and 61.8% had no apathy at baseline or follow-up. The proportion of patients having apathy symptoms did not change in the fluoxetine group (McNemar χ^2^ = 0.69, p = 0.41). In the placebo group, 7.6% of patients had apathy at baseline and follow-up, 9.6% had apathy at baseline, but not follow-up, 21.4% had no apathy at baseline, but had apathy at follow-up, and 61.4% had no apathy at baseline or follow-up. The proportion of patients having apathy symptoms increased in the placebo group (McNemar χ^2^ = 30.05, p ⩽ 0.00001).

In the fluoxetine group, 0.7% of patients had anhedonia at baseline and follow-up, 5.9% had anhedonia at baseline, but not at follow-up, 7.6% had no anhedonia at baseline, but did at follow-up, and 85.8% had no anhedonia at baseline or follow-up. Patients endorsing anhedonia did not change in the fluoxetine group (McNemar χ^2^ = 1.32, p = 0.25). In the placebo group, 0.9% of patients had anhedonia at baseline and follow-up, 6.1% had anhedonia at baseline, but not at follow-up, 6.7% had no anhedonia at baseline, but did at follow-up, and 86.3% had no anhedonia at baseline or follow-up. Patients endorsing anhedonia did not change in the fluoxetine group (McNemar χ^2^ = 0.10, p = 0.75).

## Discussion

In a post hoc analysis of a large randomized controlled trial, apathetic and depressive symptoms responded differently to fluoxetine treatment post-stroke. Apathetic symptoms increased in a similar fashion over time in both the fluoxetine and placebo groups. In contrast, depressive symptoms reduced from baseline to 6 months in the fluoxetine, but not placebo, group. These findings suggest that fluoxetine reduces post-stroke depressive symptoms, but do not alter the time course of apathetic symptoms post-stroke. These differences were not observed when examining total MADRS score, highlighting the importance of dissociating apathetic and depressive symptoms when examining treatment effects.

Apathetic symptoms increased over time in both groups consistent with some studies reporting increased apathy over time following stroke.^15^ In addition, there was an increase in the proportion of patients reporting apathy symptoms after 6 months in the placebo group. This was not observed in the fluoxetine group, although this may be explained by that group having higher rates of apathy at baseline. These results suggest that post-stroke patients experience a greater burden of motivational deficits over time, although overall severity may be mild. Research on 1 year trajectories suggests that the majority of post-stroke patients can be categorized into high (7%), moderate (33%), or low/no (50%) levels of apathy from the acute phase, which remains stable for up to a year.^16^ A small group of patients will spontaneously improve (7%) or worsen (7%).^14^ Over the course of 5 years following stroke, the prevalence of apathy was reported to increase by about 10%.^15^ Theoretical work suggests that increases in post-stroke apathy can be explained by anterograde neurodegeneration, which can propagate from the initial infarct to large-scale brain networks underlying motivation.^17^ These networks may also be affected by ischemic white matter disease,^2,18^ which was supported by our finding that apathy increased in the SVD subgroup.

Decreases in depressive symptoms in the fluoxetine group were observed in patients with age ⩽70 years, SVD, and NIHSS ⩽5, suggesting that fluoxetine may be more effective in treating depressive symptoms in stroke patients with less severe disease. Why this may be the case is unclear. One explanation could be that the pathogenesis and development of depressive symptoms are different in SVD compared to other stroke types, and that these are more amenable to SSRI treatment. Regardless of the reasons, future treatment studies should replicate and further investigate the possibility that fluoxetine can alleviate depressive symptoms in participants with mild disease. It is important to emphasize that these claims pertain to symptomatic, rather than syndromic, depression (e.g. major depression). All patients enrolled in EFFECTS were free from depression upon entry to the trial. Therefore, the decrease in depressive symptoms observed in the fluoxetine group should be interpreted as a decrease in subthreshold depressive symptomatology, rather than the treatment of a pre-existing depressive disorder.

The depressive construct we investigated excluded anhedonia. Research suggests that SSRIs can ameliorate depressive symptomatology *in general*, but specific effects for treating anhedonia are mixed.^19^ Our results supported this, with anhedonic symptoms not differing between groups or decreasing in the fluoxetine group over time. We did, however, observe an increase in anhedonia in the placebo group. Theoretical work suggests that apathy and anhedonia may increase over time due to similar pathophysiological mechanisms.^6^ If this is the case, then it is possible that fluoxetine prevented an increase in anhedonia in patients who received it.

A strength of this study is that it used a large randomized controlled trail data set. A limitation is that it was a post hoc analysis. Rather than using individual scales for apathy and depression, both measures had to be derived from the MADRS. Our results should therefore be replicated in future studies, which can be designed around assessing fluoxetine-related changes in apathy and depression as a primary outcome. Three large trials have examined the effect of fluoxetine post-stroke, but only the EFFECTS trial had data collected which allowed comparison of fluoxetine on apathetic and depressive symptoms.^20^ This study also has a number of limitations primarily related to the tools we had available to assess apathy. Apathy and anhedonia were only assessed using a single item. This limits our conclusions to symptomatic apathy and anhedonia, rather than broader syndromes. Item 7 of the MADRS, which we used to assess apathy, primarily measures slowness or difficulty initiating activities and therefore is best seen as an index of behavioral apathy, but is not a good indicator of the motivation aspect of apathy. The use of single items may also reduce sensitivity to detect effect; of note, depressive symptoms were in contrast assessed using eight items. Furthermore, we cannot make conclusions about the apathy severity from the one item we used. Another related limitation is that recent studies have suggested apathy is a multidimensional concept, including different facets, such as cognitive, behavioral, and emotional.^21^ The use of a single item for apathy did not allow us to examine differences on particular dimensions of apathy. In addition, previous work examining a three-item measure of apathy derived from the Geriatric Depression Scale-15 revealed that the measure had low sensitivity and high specificity.^22^ This suggests that the use of a single-item ad hoc measure of apathy in this study may have led to an underestimation of the prevalence of apathy in our sample.

In conclusion, we have shown that fluoxetine has differential effects on post-stroke apathetic and depressive symptoms in a post hoc analysis of a large randomized trial data set. An increase in apathetic symptoms was observed in both groups, suggesting that fluoxetine is ineffective in treating motivational deficits. In contrast, depressive symptoms decreased in the fluoxetine group, suggesting a possible treatment effect. Our preliminary results suggest that alternative strategies, both pharmacological and behavioral, for treating apathy after stroke need to be developed.

## Supplemental Material

sj-docx-1-wso-10.1177_17474930221124760 – Supplemental material for Does fluoxetine reduce apathetic and depressive symptoms after stroke? An analysis of the Efficacy oF Fluoxetine—a randomized Controlled Trial in Stroke trial data set Click here for additional data file. Supplemental material, sj-docx-1-wso-10.1177_17474930221124760 for Does fluoxetine reduce apathetic and depressive symptoms after stroke? An analysis of the Efficacy oF Fluoxetine—a randomized Controlled Trial in Stroke trial data set by Jonathan Tay, Björn Mårtensson, Hugh S Markus and Erik Lundström in International Journal of Stroke
