# Supplementary material for: Does fluoxetine reduce apathetic and depressive symptoms after stroke? An analysis of the Efficacy oF Fluoxetine—a randomized Controlled Trial in Stroke trial data set
Source: Int J Stroke. 2022 Sep 19;18(3):285–95. doi: 10.1177/17474930221124760 (PMC9940155; doi:10.1177/17474930221124760)
Supplement: sj-docx-1-wso-10.1177_17474930221124760 – Supplemental material for Does fluoxetine reduce apathetic and depressive symptoms after stroke? An analysis of the Efficacy oF Fluoxetine—a randomized Controlled Trial in Stroke trial data set [file sj-docx-1-wso-10.1177_17474930221124760.docx]

**Supplementary Table 1.** Baseline characteristics of participants included and excluded from the main analysis in EFFECTS.

|  | Included (n = 1369) | Excluded (n = 131) | P |
| --- | --- | --- | --- |
| Age, mean (SD) | 71.1 (10.8) | 73.6 (11.5) | 0.003 |
| Sex, female, n (%) | 523 (38.2%) | 50 (38.2%) | 1.00 |
| NIHSS, median (IQR) | 3.0 (2-6) | 5.0 (3-9) | 0.00003 |
| Stroke type |  |  | 0.36 |
| Ischemic, n (%) | 1194 (87.2%) | 115 (87.8%) |  |
| Hemorrhagic, n (%) | 173 (12.6%) | 12 (9.2%) |  |
| Ischemic stroke cause* |  |  | <0.00001 |
| Large artery disease, n (%) | 176 (12.9%) | 16 (12.2%) |  |
| Small vessel disease, n (%) | 395 (28.9%) | 25 (19.1%) |  |
| Cardioembolism, n (%) | 254 (18.6%) | 49 (37.4%) |  |
| Other, n (%) | 39 (2.8%) | 2 (1.5%) |  |
| Unknown or uncertain, n (%) | 336 (24.5%) | 20 (15.3%) |  |
| Treatment |  |  | 0.58 |
| Fluoxetine, n (%) | 681 (49.7%) | 69 (52.7%) |  |
| Placebo, n (%) | 688 (50.3%) | 62 (47.3%) |  |

*Note*. NIHSS = National Institute of Health Stroke Scale. * = assessed using modified TOAST criteria.

**Supplementary Table 2**. Longitudinal subgroup comparisons within fluoxetine and placebo groups unadjusted for the false discovery rate.

|  |  | Fluoxetine | | | | Placebo | | | |  |
| --- | --- | --- | --- | --- | --- | --- | --- | --- | --- | --- |
|  |  | n | Baseline | 6 months | P | n | Baseline | 6 months | P |  |
| **Age** | |  |  |  |  |  |  |  |  |  |
| >70 | Total | 405 | 2.66 | 2.82 | 0.71 | 383 | 2.72 | 2.95 | 0.4 |  |
|  | Apathy | 405 | 0.38 | 0.62 | 0.00005 | 383 | 0.3 | 0.49 | 0.006 |  |
|  | Depression | 405 | 1.99 | 1.84 | 0.22 | 383 | 2.12 | 2.12 | 0.16 |  |
|  | Anhedonia | 405 | 0.09 | 0.13 | 0.11 | 383 | 0.08 | 0.11 | 0.35 |  |
| <=70 | Total | 276 | 3.07 | 2.51 | 0.06 | 303 | 2.61 | 3.08 | 0.39 |  |
|  | Apathy | 276 | 0.38 | 0.63 | 0.0007 | 303 | 0.29 | 0.56 | 0.0002 |  |
|  | Depression | 276 | 2.29 | 1.61 | 0.0008 | 303 | 2.01 | 2.07 | 0.75 |  |
|  | Anhedonia | 276 | 0.12 | 0.11 | 0.72 | 303 | 0.07 | 0.16 | 0.01 |  |
| **Sex** | |  |  |  |  |  |  |  |  |  |
| Female | Total | 270 | 3.07 | 2.79 | 0.23 | 253 | 2.48 | 2.83 | 0.45 |  |
|  | Apathy | 270 | 0.4 | 0.54 | 0.05 | 253 | 0.25 | 0.51 | 0.001 |  |
|  | Depression | 270 | 2.3 | 1.87 | 0.02 | 253 | 1.88 | 1.94 | 0.81 |  |
|  | Anhedonia | 270 | 0.11 | 0.13 | 0.7 | 253 | 0.1 | 0.15 | 0.31 |  |
| Male | Total | 411 | 2.66 | 2.63 | 0.85 | 433 | 2.79 | 3.11 | 0.62 |  |
|  | Apathy | 411 | 0.37 | 0.68 | <0.00001 | 433 | 0.32 | 0.53 | 0.001 |  |
|  | Depression | 411 | 1.99 | 1.67 | 0.03 | 433 | 2.18 | 2.19 | 0.17 |  |
|  | Anhedonia | 411 | 0.09 | 0.12 | 0.35 | 433 | 0.06 | 0.12 | 0.02 |  |
| **Stroke type** | |  |  |  |  |  |  |  |  |  |
| Ischemic | Total | 599 | 2.82 | 2.64 | 0.4 | 595 | 2.62 | 2.92 | 0.82 |  |
|  | Apathy | 599 | 0.39 | 0.62 | <0.00001 | 595 | 0.28 | 0.5 | 0.00002 |  |
|  | Depression | 599 | 2.1 | 1.7 | 0.003 | 595 | 2.05 | 2.05 | 0.19 |  |
|  | Anhedonia | 599 | 0.1 | 0.13 | 0.31 | 595 | 0.07 | 0.13 | 0.02 |  |
| Hemorrhagic | Total | 82 | 2.88 | 3.12 | 0.7 | 91 | 3.04 | 3.54 | 0.63 |  |
|  | Apathy | 82 | 0.34 | 0.7 | 0.007 | 91 | 0.41 | 0.63 | 0.11 |  |
|  | Depression | 82 | 2.22 | 2.07 | 0.3 | 91 | 2.24 | 2.38 | 0.84 |  |
|  | Anhedonia | 82 | 0.09 | 0.1 | 1 | 91 | 0.11 | 0.16 | 0.52 |  |
| **Ischemic stroke type** | |  |  |  |  |  |  |  |  |  |
| Large artery disease | Total | 95 | 2.56 | 3.08 | 0.06 | 81 | 2.91 | 3.73 | 0.32 |  |
|  | Apathy | 95 | 0.36 | 0.68 | 0.03 | 81 | 0.37 | 0.56 | 0.27 |  |
|  | Depression | 95 | 1.95 | 1.93 | 0.89 | 81 | 2.27 | 2.62 | 0.65 |  |
|  | Anhedonia | 95 | 0.07 | 0.22 | 0.07 | 81 | 0.07 | 0.25 | 0.05 |  |
| Small vessel disease | Total | 199 | 2.88 | 2.61 | 0.19 | 196 | 2.07 | 2.64 | 0.09 |  |
|  | Apathy | 199 | 0.39 | 0.65 | 0.004 | 196 | 0.22 | 0.45 | 0.009 |  |
|  | Depression | 199 | 2.21 | 1.69 | 0.01 | 196 | 1.66 | 1.9 | 0.48 |  |
|  | Anhedonia | 199 | 0.1 | 0.1 | 0.99 | 196 | 0.04 | 0.11 | 0.03 |  |
| Cardioembolism | Total | 120 | 2.71 | 2.19 | 0.11 | 134 | 2.87 | 3.37 | 0.58 |  |
|  | Apathy | 120 | 0.29 | 0.58 | 0.001 | 134 | 0.32 | 0.6 | 0.007 |  |
|  | Depression | 120 | 2.02 | 1.39 | 0.02 | 134 | 2.17 | 2.34 | 0.63 |  |
|  | Anhedonia | 120 | 0.12 | 0.07 | 0.4 | 134 | 0.12 | 0.15 | 0.67 |  |
| Other | Total | 25 | 2.8 | 3.04 | 0.96 | 14 | 4.14 | 2.93 | 0.22 |  |
|  | Apathy | 25 | 0.16 | 0.48 | 0.14 | 14 | 0.14 | 0.07 | 1 |  |
|  | Depression | 25 | 2.44 | 2.32 | 0.69 | 14 | 3.36 | 2.43 | 0.38 |  |
|  | Anhedonia | 25 | 0.12 | 0.08 | 0.85 | 14 | 0.14 | 0 | 0.35 |  |
| Unknown | Total | 164 | 2.52 | 2.61 | 0.77 | 172 | 2.77 | 2.42 | 0.008 |  |
|  | Apathy | 164 | 0.4 | 0.52 | 0.14 | 172 | 0.28 | 0.44 | 0.07 |  |
|  | Depression | 164 | 1.79 | 1.76 | 0.96 | 172 | 2.15 | 1.67 | 0.001 |  |
|  | Anhedonia | 164 | 0.08 | 0.12 | 0.28 | 172 | 0.08 | 0.08 | 0.92 |  |
| **NIHSS** | |  |  |  |  |  |  |  |  |  |
| >5 | Total | 211 | 2.38 | 2.7 | 0.32 | 188 | 2.74 | 2.77 | 0.67 |  |
|  | Apathy | 211 | 0.3 | 0.7 | <0.00001 | 188 | 0.32 | 0.45 | 0.15 |  |
|  | Depression | 211 | 1.84 | 1.66 | 0.27 | 188 | 2.05 | 1.95 | 0.39 |  |
|  | Anhedonia | 211 | 0.05 | 0.12 | 0.07 | 188 | 0.1 | 0.12 | 0.7 |  |
| <=5 | Total | 470 | 3.02 | 2.69 | 0.08 | 498 | 2.65 | 3.09 | 0.86 |  |
|  | Apathy | 470 | 0.42 | 0.59 | 0.001 | 498 | 0.28 | 0.55 | 0.00001 |  |
|  | Depression | 470 | 2.23 | 1.79 | 0.002 | 498 | 2.08 | 2.15 | 0.33 |  |
|  | Anhedonia | 470 | 0.12 | 0.12 | 0.94 | 498 | 0.07 | 0.14 | 0.007 |  |
| **Medication adherence** | |  |  |  |  |  |  |  |  |  |
| Adherent | Total | 542 | 2.78 | 2.56 | 0.19 | 550 | 2.74 | 3.2 | 0.75 |  |
|  | Apathy | 542 | 0.39 | 0.62 | 0.00001 | 550 | 0.3 | 0.56 | <0.00001 |  |
|  | Depression | 542 | 2.07 | 1.63 | 0.0006 | 550 | 2.13 | 2.21 | 0.31 |  |
|  | Anhedonia | 542 | 0.1 | 0.12 | 0.45 | 550 | 0.08 | 0.15 | 0.01 |  |
| Not adherent | Total | 138 | 3.01 | 3.22 | 0.68 | 135 | 2.42 | 2.24 | 0.47 |  |
|  | Apathy | 138 | 0.36 | 0.62 | 0.002 | 135 | 0.28 | 0.36 | 0.36 |  |
|  | Depression | 138 | 2.28 | 2.23 | 0.88 | 135 | 1.87 | 1.65 | 0.42 |  |
|  | Anhedonia | 138 | 0.09 | 0.12 | 0.57 | 135 | 0.05 | 0.07 | 0.74 |  |
